# How “Pharmacoresistant” is Ca_v_2.3, the Major Component of Voltage-Gated R-type Ca^2+^ Channels?

**DOI:** 10.3390/ph6060759

**Published:** 2013-05-27

**Authors:** Toni Schneider, Maxine Dibué, Jürgen Hescheler

**Affiliations:** 1 Institute of Neurophysiology, University of Cologne, Robert-Koch-Str. 39, Cologne D-50931, Germany; E-Mail: j.hescheler@uni-koeln.de; 2 Department for Neurosurgery, Medical Faculty, Heinrich Heine University, Moorenstraße 5, Duesseldorf D-40225, Germany & Center of Molecular Medicine, Cologne D-50931, Germany

**Keywords:** drug sensitivity, anticonvulsive drugs, experimentally induced epilepsy

## Abstract

Membrane-bound voltage-gated Ca^2+^ channels (VGCCs) are targets for specific signaling complexes, which regulate important processes like gene expression, neurotransmitter release and neuronal excitability. It is becoming increasingly evident that the so called “resistant” (R-type) VGCC Ca_v_2.3 is critical in several physiologic and pathophysiologic processes in the central nervous system, vascular system and in endocrine systems. However its eponymous attribute of pharmacologic inertness initially made in depth investigation of the channel difficult. Although the identification of SNX-482 as a fairly specific inhibitor of Ca_v_2.3 in the nanomolar range has enabled insights into the channels properties, availability of other pharmacologic modulators of Ca_v_2.3 with different chemical, physical and biological properties are of great importance for future investigations. Therefore the literature was screened systematically for molecules that modulate Ca_v_2.3 VGCCs.

## 1. The Ca_v_2.3 Voltage-Gated Ca^2+^ Channel

**Figure 1:**
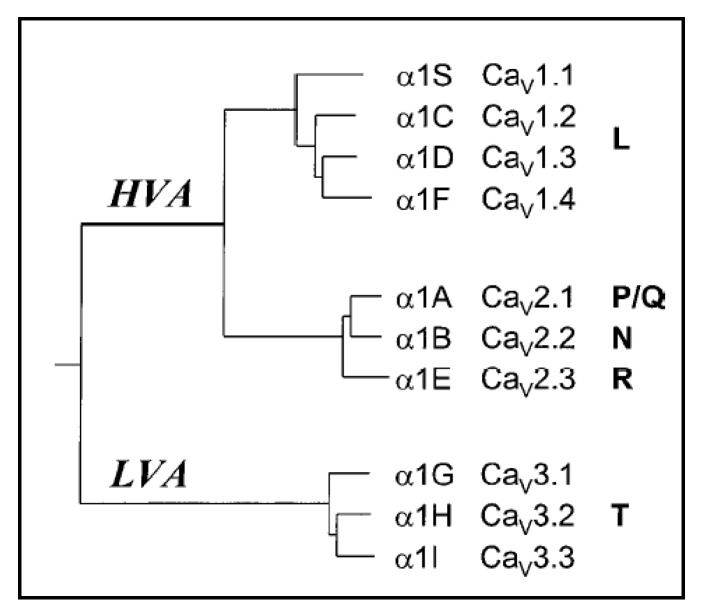
Evolutionary tree of voltage-gated Ca^2+^ channels (modified according to [5]). The cDNA of the putative membrane-spanning regions including the pore loops of the human sequences were aligned.

Ca_v_2.3 belongs to the family of voltage-gated Ca^2+^ channels which comprises ten different genes for ion conducting pore proteins (Figure 1). The ion conducting pore protein of the Ca_v_2.3 VGCCs was initially cloned from a rabbit brain cDNA library [1]. After functional expression of the rat Ca_v_2.3 clone, it was initially speculated that it may represent the low voltage-activated T-type Ca^2+^ channel, which was not yet structurally identified at that time [2]. However, consecutive cloning and expression of human Ca_v_2.3 splice variants in *X. laevis* oocytes or HEK-293 cells revealed a VGCC with properties closer resembling a high-voltage-gated Ca^2+^ channel [3,4].

Although the structure of Ca_v_2.3 deduced from sequencing of cDNA has now been known for several years [6,7], its physiological and patho­physiological roles are far from fully understood [8,9,10]. Evolutionarily, Ca_v_2.3 may have developed very early [5,11], which may underline its great significance *in vivo*. The total quaternary structure of a Ca_v_2.3-containing native VGCC is still unknown, but may contain additional subunits including the well known auxiliary β-subunits, which have been shown to modulate Ca_v_2.3-mediated inward currents in heterologous expression systems [12,13]. Molecular properties of Ca_v_2.3 have been characterized on the amino acid level for functional protein-protein interaction [14,15,16] however to date, Ca_v_2.3 VGCCs have yet to be purified as has been done for L-type Ca^2+^ channels from rabbit skeletal muscle [17,18,19,20], and bovine heart [21] and for the neuronal N-type Ca^2+^ channels [22,23].

Sequence comparison of the deduced primary sequence revealed the well known intramolecular homology pattern, which is known for all voltage-gated Ca^2+^ as well as for voltage-gated Na^+^ channels. It contains four internal repeats, which have been termed domains I, II, III, and IV. Secondary structure analysis predicts 6 transmembrane segments including a random coiled short part between transmembrane segment 5 and 6, the pore forming segment (P-loop) [24]. Many of these structure predictions resemble the confirmed structural elements in the bacterial and rat voltage-gated K^+^-channel [25,26] and a bacterial Na^+^-channel [27,28].

Additional elements may contribute to the kinetic properties of Ca_v_2.3-mediated inward currents as reported for structurally similar ion channels. The segments S6 participate in gating the ion channels [29,30,31,32], and the P-loops form essential parts of the selectivity filters, thereby also influencing the speed of the ion flux through the pore [33,34,35,36,37,38,39,40]. The segment S4 acts mainly as the voltage sensor [41,42], and its detailed orientation to the pore region has been elucidated in crystals from the bacterial K^+^ channel to a great extent [43].

Only segments of the cytosolic loops from Ca_v_1.2 L-type VGCCs have been co-crystallized with functionally auxiliary subunits [44] or functionally interacting calmodulin [45,46,47,48]. Few protein interactions of Ca_v_2.3 have been reported such as with a β-subunit [15,16] or with novel partners in heterologous expression systems [49,50,51,52], however, they have yet to be investigated by crystallization. The β-subunit interaction site with Ca_v_1.1 and Ca_v_1.2 is located in a conserved region between domain I and II [53,54], which also contains the inter­action site of Ca_v_2.3 with β-subunits [14,15,16].

The II-III linker harbors a unique site located within the arginine-rich stretch, which is responsible for Ca^2+^-mediated modulation of the Ca_v_2.3 voltage-gated Ca^2+^ channel [55]. It may be involved in the protein kinase C (PKC)-mediated signaling to Ca_v_2.3 [56], linking Ca_v_2.3 signaling to muscarinic receptor activation [57,58,59,60,61] and perhaps also to muscarinic enhancement of the “toxin-resistant” R-type Ca^2+^ current in hippocampal CA1 pyramidal neurons [62]. Ca_v_2.3 also contains the better known, carboxyterminal Ca^2+^/calmodulin interaction site, which was not only found for the members of the Ca_v_2/non-L-type but also for members of the classical L-type Ca^2+^ channel subfamily [63].

Structurally, a broad set of splice variants can be predicted from the different cloning approaches (Table 1), which result from alternate use of exon 19 encoded arginine-rich segment in the II-III loop, as well as from the alternate use of exon 45 in the carboxyterminal region [7]. Ca_v_2.3d was originally cloned as a fetal splice variant from human brain [4]. Interestingly, the major splice variants (Table 2) deduced from RT-PCR studies differ between brain regions [64] in mice. Splice variants of Ca_v_2.3 from different species (see also Table 1, Table 2) as well as auxiliary subunits are tissue-specifically expressed [9]. In addition to expression in neuronal [65,66,67,68,69] and endocrine tissues [70,71,72,73,74,75,76,77,78,79,80,81,82,83,84,85], Ca_v_2.3 transcripts have also been detected in mamalian heart [86,87,88], kidney [70,86,89], sperm [90,91,92,93], spleen [3], and retina [94,95,96,97]. Furthermore, the subcellular distribution of Ca_v_2.3 has been investigated revealing both somatodendritic and presynaptic expression [98] with additional functional specificities [99].

**Table 1:** Splice variants of voltage-gated Ca_v_2.3 R-type Ca^2+^ channels. Exon 19 is encoding an arginine-rich segment of the cytosolic loop between domain II and III, which is responsible for a transient positive Ca^2+^ feedback, when cytosolic Ca^2+^ is in­creasing by Ca^2+^ influx through the channel itself. Exon 45 is encoding a carboxy­terminal insertion of unknown function. Details of exon 20 sequence are found in [7].

Nomenclature, splice variant		Structure related to alternate exons expressed (+)			Expression (tissue and species)	Ref.
Novel terms	Old terms	Exon 19 (57 nts)	Segment (21 nts) in exon 20	Exon 45 (129 nts)		
Ca_v_2.3a	alpha1E-1	-	+	-	Rat cerebellum	[100]
Ca_v_2.3b	alpha1E-2	+	-	-	Less important in CNS	[3]
Ca_v_2.3c	alpha1E-3	+	+	-	Dominant in CNS	[3]
Ca_v_2.3d	alpha1Ed	+	+	+	Human fetal brain	[4]
Ca_v_2.3e	alpha1Ee	-	+	+	Pancreas, kidney, heart	[70,101]
Ca_v_2.3f	alpha1Ef	+	-	+	Rat cerebellum	[100]

**Table 2:** Transcripts of major splice variants of voltage-gated Ca_v_2.3 R-type Ca^2+^ channels expressed in different brain regions [64].

Brain region (mouse)	Major splice variant	Miscellaneous
Neocortex	Ca_v_2.3c	Minor amounts of Ca_v_2.3e
Hippocampus	Ca_v_2.3c	Minor amounts of Ca_v_2.3e
Thalamus	Ca_v_2.3c	Substantial amounts of Ca_v_2.3e and Ca_v_2.3f
Cerebellum, mesencephalon, medulla oblongata	Ca_v_2.3e	minor amounts of Ca_v_2.3a

In heterologous expression systems, Ca_v_2.3c [3] and Ca_v_2.3d [4,102] inward currents are activated at test potentials of about −30 mV. The single channel conductance is about 14 pS [103], and the channel kinetics measured by patch-clamp recordings reveal a fast activating and inactivating channel type with transient inward current characteristics [7,55], similar but not as fast as observed for T-type voltage-gated Ca^2+^ channels [13].

## 2. Selective and Non-Selective Antagonists of Ca_v_2.3

The first “pharmacoresistant” Ca^2+^ current in vivo was recorded and published in 1993 [104,105], which means it occured between the years 1987 (the first cloning of a VGCC subunit [106]) and 1994 (final cloning of the remaining high-voltage gated Ca^2+^ channels). Doe-1, cloned from *Discopyge ommata*, represented a novel Ca^2+^ channel type, which was insensitive towards dihydropyridines, but was antagonized rather than activated by 5 µM Bay K. This chan­nel type was only slightly and readily reversibly inhibited by 5 µM ω-conotoxin-MVIIC, was insensitive towards ω-agatoxin-IVA, and fully reversibly blocked by ω-conotoxin-GVIA, an irreversible antagonist of N-type Ca^2+^ channels [104]. Interestingly, the same group identified a similar Ca^2+^ current component in rat cerebellar granule neurons and called the doe-1-like component “R-type current” [105].

The peptide antagonist SNX-482, which was initially purified from the venom of the tarantula *Hysterocratis gigas* [107] blocks Ca_v_2.3 with an IC_50_ value of 15–30 nM and was the first and still is the only Ca_v_2.3-prevalent antagonist,. At concentrations higher than 500 nM SNX-482 also inhibits N-type Ca^2+^ currents [107], wherease L-type Ca^2+^ currents are inhibited by about 25% at concentrations of 200 nM SNX-482 [108]. Therefore, it only can be regarded as Ca_v_2.3-prevalent, but not as Ca_v_2.3-specific or -selective.

In cerebellar granule cells, two Ca_v_2.3 isoforms could be distinguished from eachother by their varying SNX-482 IC_50_ values of 6 nM and 81 nM, and a third R-type Ca^2+^ current component by its insensitivity to SNX-482 [109].

The first gene inactivation of Ca_v_2.3 led to knock-out mice, which in cerebellar granule cells and in DRG neurons still expressed a drug insensitive Ba^2+^ current. The peak inward current (I_Ba_) was even larger than in cultured mouse neurons from contol mice (knock-out I_Ba_ 113 ± 27 pA (n = 5 ); control 85 ± 21 pA (n = 9)) [110]. Only the wild type cultured neurons were inhibited by SNX-482, but not the neurons from Ca_v_2.3-deficient mice, leading to the conclusion that a non-Ca_v_2.3-dependent R-type current may exist.

In murine hippocampal and neocortical neurons, Ca_v_2.3 contributes not only to the SNX-482-sensitive component of the R-type Ca^2+^ current, which was recorded in the presence of combination of Ca^2+^ channel antagonists (ω-conotoxin-GVIA, 2 µM; ω-conotoxin-MVIIC, 3 µM; ω-agatoxin-IVA, 200 nM; nifedipine, 10 µM), but also to the SNX-482-insensitive part [66]. Interestingly, the voltage of half-maximal activation (V_1/2, act_) was shifted to more positive voltages in all three cell types investigated (dissociated CA1 pyramidal cells, dentate gyrus cells, neocortical neurons), specially in the neocortex, where it was reduced from –68 ± 2 mV to –58 ± 7 mV [66]. Overall, it may be useful to keep in mind that the R-type Ca^2+^ current may be more than only the Ca_v_2.3-gene encoded Ca^2+^ channel in neuronal tissues [8,111,112].

Divalent and trivalent heavy metal cations were often used to antagonize either all voltage-gated Ca^2+^ inward currents (Cd^2+^, La^3+^) or to specifially inhibit some T-type and the R-type Ca^2+^ current (Ni^2+^). Unfortunately, the half maximal concentrations for Ca_v_2.3 and Ca_v_3.2 are close to each other (10–30 µM), rendering Ni^2+^ blockade unsuitable for distinction of Ca_v_2.3 currents in tissue in which Ca_v_3.2 is also expressed. Physiologically, homeostasis of other divalent cations like Cu^2+^ and Zn^2+^ may play an important role [10,113,114], notably also in neurodegenerative disease [115].

Table 3 summarizes the effect of drugs and toxins on Ca_v_2.3 reported in the literature. Most drugs in the table are non-selective, in the sense that currents through other Ca^2+^ channel Ca_v_α1 subunits are also antagonized with an IC_50_ not larger than tenfold. Many substances show inhibitory effects on Ca_v_2.3 or on R-type Ca^2+^ currents. One set of drugs is related to anticonvulsive effects, others are used as anesthetic drugs. Even high concentrations of classical Ca^2+^ channel antagonists can inhibit Ca_v_2.3 induced inward currents as shown for the dihydropyrdines isradipine [87] and nicardipine [116]. Routinely, in order to block L-type voltage-gated Ca^2+^ channels, a dihydropyridine concentration of around 10 µM is chosen by electrophysiologists. Considering that such high concentrations of isradipine or nicardipine substantially block E-/R-type Ca^2+^ currents, lower concentrations of e.g., isradipine of 0.5 µM are more suitable, in order to observe antagonism by low concentrations of SNX-482 as shown for cardiac E-/R-type Ca^2+^ currents in murine myocytes [101]. However, one has to keep in mind that SNX-482 may block L-type Ca^2+^ current at elevated concentrations [108].

## 3. Physiological Functions, in Which Ca_v_2.3 may be Involved, as Deduced from Ca_v_2.3-Deficient Mice

Many of the experimental results from gene-inactivated mice cannot automatically transferred to human physiology and pathophysiology of human diseases. But some basic conclusions may be drawn from these investigations of Ca_v_2.3-deficient mice, which were generated and analysed in several different laboratories (for detail, see Kamp *et al*. [8]).

Ca_v_2.3 is expressed in many regions of the CNS and also in peripheral organs and tissues, which makes it difficult to explore its full function in vivo. Ca_v_2.3 triggers or participates in the release of several neurotransmitters such as dopamine in the substantia nigra [117]. In the hippocampus Ca_v_2.3 contributes to fast glutamatergic transmission [118], where it is also involved in long term potentiation at the mossy fiber – CA3 synapses. Therefore, Ca_v_2.3 participates in basic processes related to learning and memory formation [67,119,120,121]. Furthermore, Ca_v_2.3 is an important regulator in spines: activation of Ca_v_2.3 triggers opening of small conductance Ca^2+^-activated K^+^-channels in CA1 hippocampal pyra­midal neurons [122,123,124], suggesting spine-restricted local microdomains, which are important for synaptic signalling [125]. R-type Ca^2+^ currents, which were recorded as Ni^2+^-sensitive tail currents, are available at resting potential and contribute to after-depolarization, and thus to the initiation of burst firing in CA1 hippocampal neurons [126].

**Table 3:** Selected antagonists of Ca_v_2.3 (modified according to: Wrubel, 2009 [127]). Recombinant Ca_v_2.3 was expressed in different cell lines and was cotransfected with auxiliary subunits (β-subunits from different species). Note, trace metals must be applied under well defined conditions, which provide buffering of the cation of interest [10]. Abbreviations: n.t. = not tested.

Substance	Application	IC_50_ or K_d_ [µM]	Amount of max. Inhibition	Selectivity	Ref.
SNX-482	Peptide toxin	0.015–0.030	> 80 %	Ca_v_2.3-prevalent	[107,108,128,129,130]
ω-Aga-IVA	Peptide toxin	0.051	80%	non-selective	[116]
ω-Aga-IIIA	Peptide toxin	0.003–0.010	100%	non-selective	[107]
Ni^2+^	Unphysiological	27.4/303	100%	non-selective	[3,131]
Cd^2+^	Unphysiological	0.8	100%	non-selective	[3]
Zn^2+^	Trace element	31.8	>90%	non-selective	[132]
Zn^2+^ (calibrated)	Trace element	1.3	100%	non-selective	[10]
Cu^2+^	Trace element	0.018	100%	non-selective	[10]
Topiramate	Anticonvulsive	50.9	>70%	non-selective	[133]
Lamotrigine	Anticonvulsive	>10		non-selective	[134]
Sipatrigine	Anticonvulsive	10	100%	non-selective	[134]
202W92	Anticonvulsive	56	100%		[134]
Ethosuximide	Anticonvulsive	20000	100%	non-selective	[135]
MPS (α-methyl­phenylsuccinimide)	Anticonvulsive	2300	100%		[135]
Phenytoin	Anticonvulsive	360	100%		[135]
Phenobarbital	Anticonvulsive	2700	>80%		[135]
Pentobarbital	Anticonvulsive	600	100%		[135]
Halothane	Inhalation anaesthetic				[136,137]
Isoflurane	Inhalation anaesthetic	206	100%		[136,138,139]
Fomocaine	Local anaestetic	95	100%		[140]
Procaine	Local anaestetic				[140]
Octanol	Organic solvent	206	100%		[135]
(+)-ACN	Steroid anaestetic	5.3–10.2	100%		[141]
(+)-ECN	Steroid anaestetic	9.9–16.1	>70%		[141]
Flecainide	Antiarrhythmic	320			[140]
Penfluridol	Antipsychotic	13			[140]
Verapamil	Antihypertensive	100	100%	non-selective	[142]
Diltiazem	Antihypertensive	220	100%	non-selective	[4,142]
Isradipine	Antihypertensive	9.1	100%	non-selective	[87]
Nicardipine	Antihypertensive	1	n.t.	non-selective	[116]
Mibefradil	Antihypertensive	0.4/6.5	100%	non-selective	[143]
Amiloride	Diuretic	7400	100%	non-selective	[135]
Ethoxyzolamide	Carboanhydrase inhibitor/anticonvulsive	1	70%		[144]
Eugenol	Analgetic				[145]
Bisphenol A	Environmental pollutant	26	50%	non-selective	[146]

The existance of a fetal brain Ca_v_2.3 isoform [4] and the changes in expression of Ca_v_2.3 during neuronal development point to an important role of Ca_v_2.3 during early prenatal stages [147,148,149]. At nerve terminals of the calyx of Held, N- and R-type Ca^2+^ channels are replaced by P-/Q-type Ca^2+^ channels during development [150].

Ca_v_2.3-deficient mice reveal altered pain response [151], and transcripts of two different splice variants of Ca_v_2.3 could be identified in rat nociceptive neurons [152]. The major splice variant was Ca_v_2.3e, which was also detected in the cerebellum, heart and endocrine system (Table 1, Table 2).

Ca_v_2.3 is highly expressed in the amygdala, in which the R-type Ca^2+^ current represents the largest component of high-voltage gated Ca^2+^ currents. Ca_v_2.3-deficient mice exhibited signs of enhanced fear assuming that Ca_v_2.3-based R-type Ca^2+^ currents in the amygdala may be associated with fear [153].

Ca_v_2.3-deficient mice represent an important model for convulsive and non-convulsive seizures as was summarized in [9]. Based on the initial detection of Ca_v_2.3 transcripts in the insulinoma cell line INS-1 [70,73], additional investigations were performed with Ca_v_2.3-deficient mice, which showed disturbance not only of glucose-induced insulin release [72,75], but also of glucose-mediated glucagon suppression [74], and more important even disturbances of glucose-mediated somatostatin-release [80].

SNX-482 sensitive R-type Ca^2+^ current was related to the release of gonadotropin-releasing hormone [81] and of oxytocin [76,77]. Overall, peptide hormone release often appears to be triggered by Ca_v_2.3 VGCCs, possibly by producing the global increase in cytoxolic Ca^2+^ required for refilling of the readily releasable pool of granules during the second phase of insulin release [75,154].

After cerebral aneurysm rupture and subarachnoidal hemorrhage Ca_v_2.3 has been shown to contribute to cerebral artery constriction *i.e.*, vasospasm [155], a devastating delayed event causing often fatal strokes. Accordingly intracisternal administration of SNX-482 reduced delayed vasospasm in a rat model of subarachnoid hemmorhage [156].

The expression of Ca_v_2.3 in cardiomyocytes is still under discussion: Ca_v_2.3 protein has yet to be detected reliably in murine cardiomyocytes, but transcripts could be amplified by single cell RT-PCR from microscopically identified murine cardiomyocytes [87,88]. Furthermore, Ca_v_2.3 ablation causes cardiac arhythmia and disturbances in autonomic cardiac control, suggesting that Ca_v_2.3 in pacemaker cells as well as in autonomic nerve endings may participate in cardiac signalling [101].

In future, more specific Ca_v_2.3 modualtors will be a key in establishing the exact role of Ca_v_2.3 in the physiological and pathophysiological processes, that it contributes to. Furthermore, recent evidence points to Ca_v_2.3 as a potential pharmacologic target in therapy of epilepsy, chronic pain, endocrine disturbances and vasospasms after subarchnoid hemmorhage. In this light, non-ion selective Ca_v_2.3 inhibitors with favourable pharmakokinetics could represent new therapeutic strategies for these disorders.
